# Performance of Dye Removal from Single and Binary Component Systems by Adsorption on Composite Hydrogel Beads Derived from Fruits Wastes Entrapped in Natural Polymeric Matrix

**DOI:** 10.3390/gels8120795

**Published:** 2022-12-03

**Authors:** Cristina-Gabriela Grigoraș, Andrei-Ionuț Simion, Lidia Favier, Cătălin Drob, Lucian Gavrilă

**Affiliations:** 1 Department of Chemical and Food Engineering, Faculty of Engineering, “Vasile Alecsandri” University of Bacău, Calea Mărășești 157, 600115 Bacău, Romania; 2 Ecole Nationale Supérieure de Chimie de Rennes, University of Rennes, CNRS, UMR 6226, CEDEX 7, 35708 Rennes, France; 3 Department of Engineering and Management, Mechatronics, Faculty of Engineering, “Vasile Alecsandri” University of Bacău, Calea Mărășești 157, 600115 Bacău, Romania

**Keywords:** cherry stones, chitosan, dye adsorption, Acid Red 66, Reactive Black 5, binary component system, response surface methodology, kinetic study, equilibrium isotherms

## Abstract

The treatment of contaminated water is currently a major concern worldwide. This work was directed towards the preparation of a composite hydrogel by entrapping cherry stones powder on chitosan, which is known as one of the most abundant natural polymers. The synthesized material was characterized by scanning electron microscopy, by Fourier transform infrared spectroscopy, and by the point of zero charge determination. Its ability to remove two azo dyes models (Acid Red 66 and Reactive Black 5) existing in single form and in binary mixture was evaluated. Response Surface Methodology–Central Composite Design was used to optimize three parameters affecting the process while targeting the lowest final contaminant concentrations. The best results were obtained at pH 2, an adsorbent dose of 100 g/L, and a temperature of 30 °C, when more than 90% of the pollutants from the single component systems and more than 70% of those of the binary mixtures were removed from their aqueous solutions. The adsorption process was in accordance with Elovich and pseudo-second-order kinetic models, and closely followed the Freundlich and Temkin equilibrium isotherms. The obtained results led to the conclusion that the prepared hydrogel composite possesses the ability to successfully retain the target molecules and that it can be considered as a viable adsorbent material.

## 1. Introduction

With the continuous increase in demand for different products, industrialization in the recent decades has resulted in the use of more and more chemical compounds. Synthetic dyes are among the most frequently encountered of these compounds. Depending on their structure, they can be divided into several categories such as azo, vat, indigo, nitro dyes, etc., the first, azo, representing more than 60% of the market [1]. Since it is common for synthetic dyes to be used in the production of goods as textiles [2,3], cosmetics [4,5,6], food [7,8,9], plastics [10], drugs [11,12], etc., they are obtained in large quantities. Part of them end up in water, either through the products in which they are found or as part of the discharged effluents thus becoming a source of contamination.

Even at very low concentrations, these pollutants, recognized as highly durable and water soluble and characterized by their own form and mechanism of action, cause serious injuries to human health and to the environment. Allergic reactions, association with systemic diseases, skin irritations [13], mutagenic and carcinogenic potential, high toxicity, respiratory and renal failure [14], cyanosis, tissue necrosis, and jaundice [15] can be cited as negative effects of dyes on humans. As well, dyes inhibit plant growth, cause reductions in pigment and protein content of microalgae, and have toxic effects on aquatic life, resulting in breathing troubles, movement difficulties, and malformations [16].

As a consequence, the removal of these emerging contaminants from wastewater has become a pressing issue and the need to maintain water quality has become a real challenge.

In order to counterbalance these growing concerns, it is imperative that dyes are removed from aqueous media at sufficiently low levels. Usually, the elimination of dyes is attempted through a variety of biological, chemical, and physical methods. The first class of treatments is typically characterized by the degradation of dyes through metabolism or adsorption by biomass (bacteria, fungi, and yeasts) before the treated aqueous effluents are released into the environment [17]. Chemical treatments include methods such as oxidation processes [18,19,20], ozonation [21,22], electrochemical destruction [23,24], photocatalysis [25,26,27], etc. In terms of physical techniques, coagulation-flocculation [28,29], membrane filtration [30], nanofiltration [31], precipitation [32,33], etc., can be cited. Even though, on occasion, satisfactory results were obtained when combinations of biological, chemical, and physical methods were used [34,35], many of these methods are ineffective for some colored compounds. Reduced degradation is observed for biological treatments. Then again, chemical and physical processes are responsible for the generation of various secondary products which are regularly classified as harmful for the environment. Moreover, due to the complex structures of dyes and their often high molecular weight, there is always a challenge in selecting the adequate approach.

As a result of the above related aspects, there is an amplified demand for the development of appropriate methodologies for the removal of dyes existing in wastewaters. Adsorption is considered to be an attractive alternative since it stands out through operation simplicity, avoidance of supplementary chemicals, and nonexistence of undesirable products [36]. Numerous adsorbents such as activated carbon [37,38,39], silicates [40], porous geopolymers [41], materials prepared from agricultural wastes [42,43] or obtained from biomass residue [44], materials containing nanoparticles of various oxides [45,46,47], composites [48,49], etc., are reported as favorable adsorbents. They possess important surface area, high pore sizes, and structures permitting high affinity with various compounds; therefore, they are listed as efficiently retaining colored contaminants from aqueous media.

Considering the actual context of the continuing efforts towards developing new methods for water depollution, the novelty of the present paper resides in using an under-valorized by-product from fruits processing industrial sector to produce an adsorbent material and revealing that it is able to retain colored molecules from aqueous solution.

Thus, the present research was undertaken to prepare a low-cost adsorbent by a simple method of entrapping a powder of cherry stones in a chitosan polymeric matrix. Morphological analysis using scanning electron microscopy (SEM), Fourier Transform Infrared Spectroscopy (FTIR) analysis, and determination of the point of zero charge (pH_PZC_) were used for its characterization. Subsequently, the adsorption potential of the obtained value-added material was evaluated for the removal of two model azo dyes: Acid Red 66 (AR) and Reactive Black 5 (RB), which are found in single or in binary component systems. Then, a Response Surface Methodology (RSM) analysis was conducted on several parameters which affect the adsorption process (pH, adsorbent amount, and temperature). Finally, on the optimized established conditions, a kinetic study was carried out and kinetic models and equilibrium isotherms were tested and established.

## 2. Results and Discussion

### 2.1. Adsorbent Synthesis and Characterization

Cherry stones are a waste product from the fruits processing industry that is highly available; however, they are usually not considered a product of interest, and are generally discarded. Our preliminary tests revealed that cherry stones, as such, are not able to adequately retain the target contaminants. Even though there are ways of using them for producing activated carbon, for example. A chemical activation process conducted with zinc chloride at 700 °C was proposed by Angin et al. [50]. Olivares-Marin et al. [51,52] used dehydration agents such as potassium hydroxide or sulfuric acid and temperatures between 400 °C and 900 °C in order to obtain activated carbon from cherry stones. Duran-Valle et al. [53] submitted cherry stones to different gases action under elevated temperature obtaining a porous material. Nowicki et al. [54] conducted a pyrolysis process on cherry stones followed by physical activation at 800 °C under carbon dioxide. The tests on the use of the prepared material for the adsorption of different pollutants were very promising. In one of our previous papers [55], we have reported, also, that the adsorbent which results from calcinating cherry stones for 6 h at 600 °C is able to retain dyes such as Congo Red from aqueous solutions. Nonetheless, all of these methods, besides being time- and energy-consuming, present the major disadvantage of a very reduced yield (only 1.2% in our earlier study).

As a consequence, in this work, we aimed, firstly, to prepare an adsorbent material by using cherry stones directly without passing them through the activation and/or calcination steps of activated carbon manufacture. Through our investigations, we have determined that, when this industrial by-product is used in a ground state, its adsorption capacity increases significantly. However, cherry stones powder is difficult to recover from an aqueous environment after the completion of the adsorption process. An alternative consists of entrapping the cherry stones into a carrier support. Chitosan (a polysaccharide derived from chitin) was chosen for this purpose. Apart from the fact that this natural polymer possesses, among others, attractive attributes such as biocompatibility, biodegradability, untoxicity, and antitumor, analgesic, antioxidant, and antimicrobial properties [56], it is known for its ability to form hydrogels.

**Figure 1:**
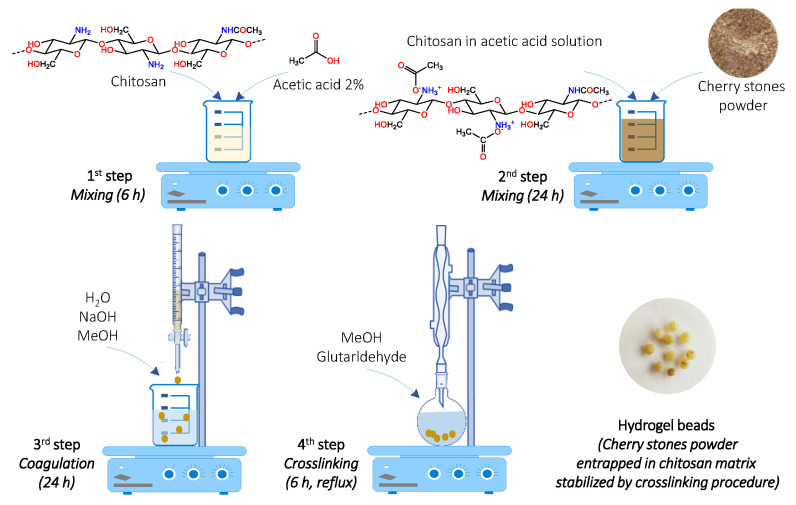
Hydrogel beads preparation procedure.

In our case, cherry stones in powder were carefully mixed for 24 h with a solution of chitosan in acetic acid. The suspension was then dripped in a methanol–sodium hydroxide combination and left to rest at 4 °C for another 24 h. The hydrogel beads were heated at reflux in a methanol–glutaraldehyde mixture for 6 h at 70 °C in order to ensure a good stability in acidic media. A schematic representation of the preparation process steps is exhibited in Figure 1.

**Figure 2:**
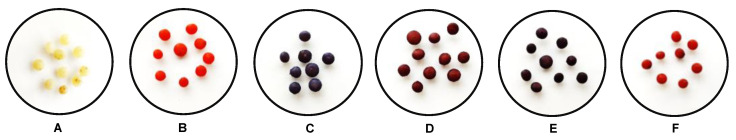
Images of CSCH beads before dye adsorption (**A**) and after adsorption of AR (**B**), RB (**C**), 25% AR + 75% RB (**D**), 50% AR + 50% RB (**E**), and 75% AR + 25% RB (**F**).

As shown in Figure 2A, the resulted beads (abbreviated CSCH) have a uniform spherical shape and a white, light yellow color with visible grains of cherry stones powder. Their mean diameter was 2.5 mm ± 0.2 mm.

**Figure 3:**
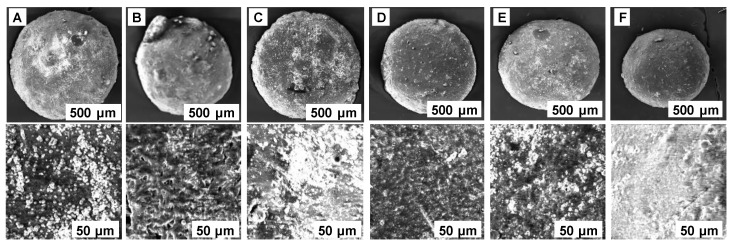
SEM photographs of CSCH beads before dye adsorption (**A**) and after adsorption of AR (**B**), RB (**C**), 25% AR + 75% RB (**D**), 50% AR + 50% RB (**E**), and 75% AR + 25% RB (**F**).

The SEM analysis of hydrogel beads revealed a smooth external surface with some irregularities that can be due to the preparation process. At the internal level, a structure with fine pores is visible. It can be also seen that the chitosan matrix englobes particles of cherry stones powder (Figure 3A). The photographs recorded after the adsorption process (Figure 3B–E) are similar both in the case of single and binary component systems, and disclose rolling tendencies.

**Figure 4:**
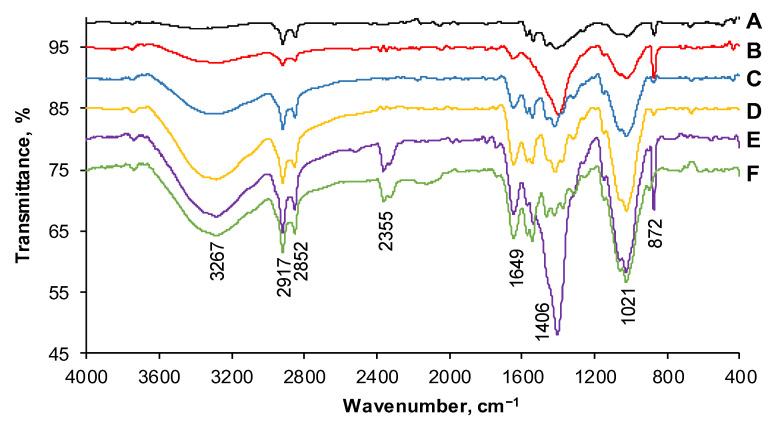
FTIR spectra of CSCH beads before dye adsorption (**A**) and after adsorption of AR (**B**), RB (**C**), 25% AR + 75% RB (**D**), 50% AR + 50% RB (**E**), and 75% AR + 25% RB (**F**).

FTIR spectra were recorded for the prepared material before and after the adsorption of the target compounds existing in the tested single or binary systems. In the case of CSCH adsorbent (Figure 4A), the band appearing between 3500 cm^−1^ and 3200 cm^−1^ was characteristic of the hydroxyl stretching vibration overlapped on the −N−H vibration of chitosan matrix.

Methylene and methylidene asymmetric stretch have a peak at approximatively 2800 cm^−1^. In the region of 1500 cm^−1^–1200 cm^−1^, bending vibrations of −N−H and of −CH_2_ can be observed [57]. At approximately 1000 cm^−1^, vibration of the C−O of the natural polymer or of cherry stones is visible [58].

The analysis of the FTIR spectra recorded after the retention of Acid Red 66 dye (Figure 4B), Reactive Black 5 (Figure 4C), or their combinations (Figure 4D–F) showed the existence of functional groups such as diazene (at about 1500 cm^−1^) or sulfonate group attached to benzene (vibrations between 1400 cm^−1^ and 1000 cm^−1^) [59] specific to AR or the bands of C−C, C=C, and N=N, which were observable from 1600 cm^−1^ to 1500 cm^−1^ [60,61] and were assignable to RB. These facts sustain the conclusion that the adsorption of the molecules took place.

The last investigation conducted for the characterization of the synthesized material was the determination of the point of zero charge (pH_PZC_). When an adsorbent is introduced in an electrolyte aqueous solution, the ions existing on its surface dissociate and form complexes with those of the electrolyte solution [62]. Hence, at different pH values, the surface charge can be negative, positive, or null, and the adsorbent acts as a cation or anion exchanger, being able to retain various molecules. A surface charge equal to 0 does not signify the absence of charge but equality between the positive and negative charges [63].

**Figure 5:**
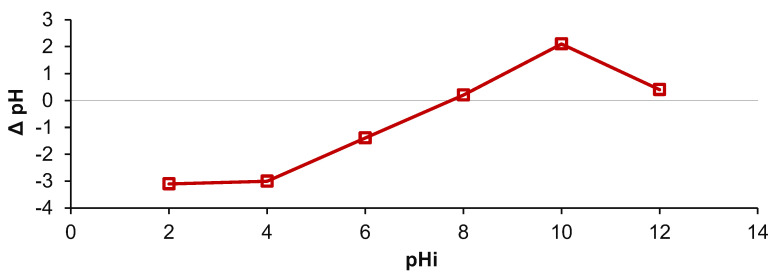
Point of zero charge of the prepared adsorbent.

From Figure 5, it can be remarked that the adsorbent obtained by entrapping cherry stones powder in chitosan has a pH_PZC_ of 7.8. Similar values were reported by other studies [54,64] in which cherry stones were used for manufacturing adsorbing materials. Theoretically, at a working pH below pH_PZC_, the hydrogel beads interact with the negative charges of the pollutants while, at a pH above the pH_PZC_, more interactions will occur with the positive charge of the chosen molecules. Practically, an increase in pH from 2 to 4 leads to an increase in pH_PZC_. The existence of a plateau is perceptible when the pH is adjusted between 4 and 10. In this interval, the adsorbent possesses amphoteric properties, meaning that neither the acid addition nor the base addition in the initial dye solution will affect the equilibrium pH.

### 2.2. Optimization of Adsorption Process Main Parameters by Response Surface Methodology

Response Surface Methodology with one of its most frequently used designs (Central Composite Design (CCD)) is an effective technique that can be applied when there are several variables influencing one or multiple responses of interest, as is the case with contaminants adsorption [65,66]. Defined as a tool which is able to explain the connections that occur between different factors in order to return optimized working conditions, RSM implies, in a first step, an appropriate development of the experimental program, including the choice of parameters influencing a response function and the set of their levels of variation. Secondly, with the acquired data, an optimization process is conducted via a software investigation. A mathematical model is generated, and its adequacy is tested by statistical analysis and confirmed by comparison with results registered when running experiments on the suggested conditions.

In these circumstances, one of our aims was to determine the optimized values for the three main parameters (pH, adsorbent dose, and temperature) affecting the adsorption of target dyes. The final contaminant concentration was the following response function.

For this study, a total of 27 experiments were conducted for each of the single dye systems (AR, RB) and for a binary system (50% AR + 50% RB).

Table 1 shows the recorded data for the response function and the values predicted by the quadratic models generated by the employed software.

The mathematical models obtained for the response function (final contaminant concentration) of each set of experiments are expressed by Equations (1)–(3).

(1) $$C_{\mathrm{AR}}=8.66+6.27\cdot X_{1}-2.08\cdot X_{2}+0.06\cdot X_{3}-0.65\cdot X_{1}\cdot X_{2}-0.29\cdot X_{1}\cdot X_{3}\;+0.33\cdot X_{2}\cdot X_{3}-0.74\cdot X_{1}^{2}+3.48\cdot X_{2}^{2}+0.92\cdot X_{3}^{2}$$

(2) $$C_{\mathrm{RB}}=10.74+8.16\cdot X_{1}-1.25\cdot X_{2}-0.23\cdot X_{3}+0.10\cdot X_{1}\cdot X_{2}-0.19\cdot X_{1}\cdot X_{3}\;+0.10\cdot X_{2}\cdot X_{3}ࢤ0.17\cdot X_{1}^{2}+3.92\cdot X_{2}^{2}+2.00\cdot X_{3}^{2}\;$$

(3) $$C_{50\%\;\mathrm{AR}+50\%\;\mathrm{RB}}=19.46+5.87\cdot X_{1}-0.22\cdot X_{2}+0.35\cdot X_{3}+0.76\cdot X_{1}\cdot X_{2}\;-0.25\cdot X_{1}\cdot X_{3}+0.04\cdot X_{2}\cdot X_{3}-2.28\cdot X_{1}^{2}+4.04\cdot X_{2}^{2}+0.43\cdot X_{3}^{2}\;$$

The interdependent consequence of the terms depends on the coefficient signs: a positive sign implies synergy and that an augmentation of the response function occurs with the increase in the factors. On the contrary, a negative sign suggests that the variable is able to conduct to a diminishment of the response function.

Statistical significance was tested by analysis of variance.

As shown in Table 2, the values of lack of fit for all three dyes systems indicate the suitability of the obtained mathematical equations. In the case of the terms, a low *p*-value suggests that the specific terms are significant. For AR and RB single systems, the pH and adsorbent dose had the most important impact while, in the case of the binary dye system, it seems that the pH had the greatest influence on the retention of the pollutants on the adsorbent surface.

The models *F*-values (260.08 for AR, 1877.61 for RB, 34.43 for 50% AR + 50% RB) imply that they are significant, with the chances of being caused by the noise reaching only 0.01%.

Correlation coefficients very close to the unit (0.9915 for AR, 0.9988 for RB, and 0.9394 for 50% AR + 50% RB) reveal that the models can satisfactorily predict the experimental results. The Predicted R^2^ (0.9784 for AR, 0.9974 for RB, and 0.8691 for 50% AR + 50% RB) were all in reasonable agreement with the Adjusted R^2^ (0.9877 for AR, 0.9983 for RB, and 0.9121 for 50% AR + 50% RB), with the difference being very reduced.

The adequate precision (which measures the signal to noise ratio) was higher than 4 for all three systems (56.810 for AR, 144.678 for RB, and 18.784 for 50% AR + 50% RB), indicating an adequate signal.

Consequently, the mathematical models can be considered as fitting for all systems.

**Figure 6:**
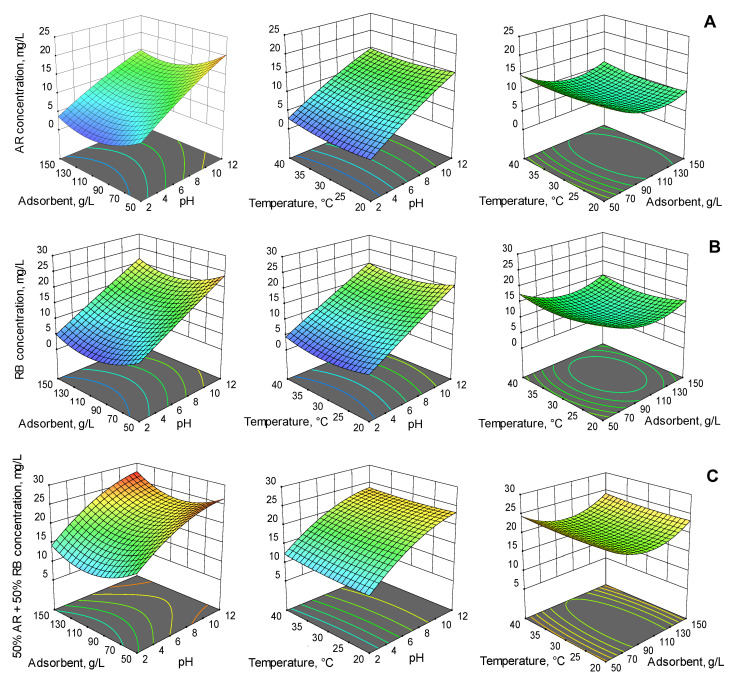
Aggregate impact of pH, adsorbent dose, and temperature on final concentration of AR (**A**), RB (**B**), and 50% AR + 50% RB (**C**).

3D response surface plots showing the factors impacts on the final dye concentration are displayed in Figure 6.

When conducting an adsorption process, the pH of the initial solution has a decisive effect. It impacts both the adsorbent properties and the characteristics of the molecules to be adsorbed [65]. In this study, more than 90% of the target compounds were removed from the aqueous solution of single dye system and more than 70% was removed when two dyes were mixed in equal proportions. The removal efficiency decreased with the augmentation of pH to neutral, and also in alkaline media. Similar data are disclosed by other papers which reveal that acid conditions are suitable for the adsorption of this type of dyes [67,68].

The amount of the adsorbent added to emerging pollutants solutions also affects the adsorption effectiveness. The most elevated values of the removal efficiency in the case of the analyzed dyes in single form or in combination were attained when the adsorbent concentration was 100 g/L. Increasing the adsorbent dose augments the probability that the active sites are occupied by the contaminants. After a certain limit (considered to be the optimal adsorbent dose), saturation is reached and no considerable increase in dye retention on the adsorbent surface is observed [69].

Temperature is another key parameter of the adsorption process. As explained by Yadav and Dasgupta [70], the increased retention of the dye chosen for their experiments when the temperature was raised from 20 °C to 30 °C could be the result of the increased dye flux getting to the adsorbent material, and of a more important diffusion rate. The decline in removal efficiency when the temperature was set at 40 °C could be the outcome of a reduced affinity of the dye to the adsorbent. Silva et al. [71] concluded that a high temperature is responsible for weakening the forces between the target molecule and the adsorbent surface, and has an undesirable impact on the process. Analogous information can be drawn from our investigation. Three temperatures were tested (20 °C, 30 °C, and 40 °C) and the best results were obtained for the middle value.

All three parameters were optimized simultaneously in order to reach the smallest contaminant concentration in the aqueous solution after the finalization of the adsorption process. The resultant values were as follows: pH 2, adsorbent dose: 100 g/L, and temperature: 30 °C. Five supplementary experiments were conducted in these conditions. The adsorption of dyes on the synthesized material was visible (Figure 2B–F). The removal efficiencies were 1.20 ± 0.05 mg/L for AR, 2.40 ± 0.1 mg/L for RB, and 7.12 ± 0.09 mg/L for 50% AR + 50% RB system, sustaining the adequacy of the mathematical models and validating them.

### 2.3. Adsorption Process Kinetic

A better understanding of the adsorption process can be achieved by studying the kinetic behavior of the adsorbent. Investigations were conducted at pH 2 and a temperature of 30 °C by putting 10 mL of dye solution in a single system (AR, RB) or binary systems (25% AR + 75% RB, 50% AR + 50% RB, and 75% AR + 25% RB), in contact with an adsorbent dose of 100 g/L. The refractory compound concentrations were established at given time intervals ranging from 10 min to 240 min.

Different known kinetic models, namely pseudo-first-order, pseudo-second-order, Elovich, and Avrami, were tested and their accuracy evaluated. The first of the mentioned models can estimate the equilibrium time and quantity of the adsorbed pollutant by determining the time-scaling factor [72]. The pseudo-second-order kinetic model states that adsorption is based on chemical relations which take place between the adsorbate and the adsorbent surface. Parameters such as initial contaminant concentration, enthalpy, and entropy changes must be considered since they have a major impact on the adsorption [73]. There are studies specifying that, for high initial concentrations, the pseudo-first-order model is more suitable to express the adsorption kinetics, while the pseudo-second-order equation is recommended for defining the adsorption conduct at low concentrations [74,75]. The Elovich equation hypothesis is related to the fact that the chemical mechanism led the process, and is appropriate for situations including adsorbents with heterogeneous surfaces [76]. The Avrami kinetic model proposes that the reaction between the adsorbate and adsorbent occurs at the active sites surface. One of its factors gives details on the changes in the adsorption mechanisms as a function of time and temperature [77].

**Figure 7:**
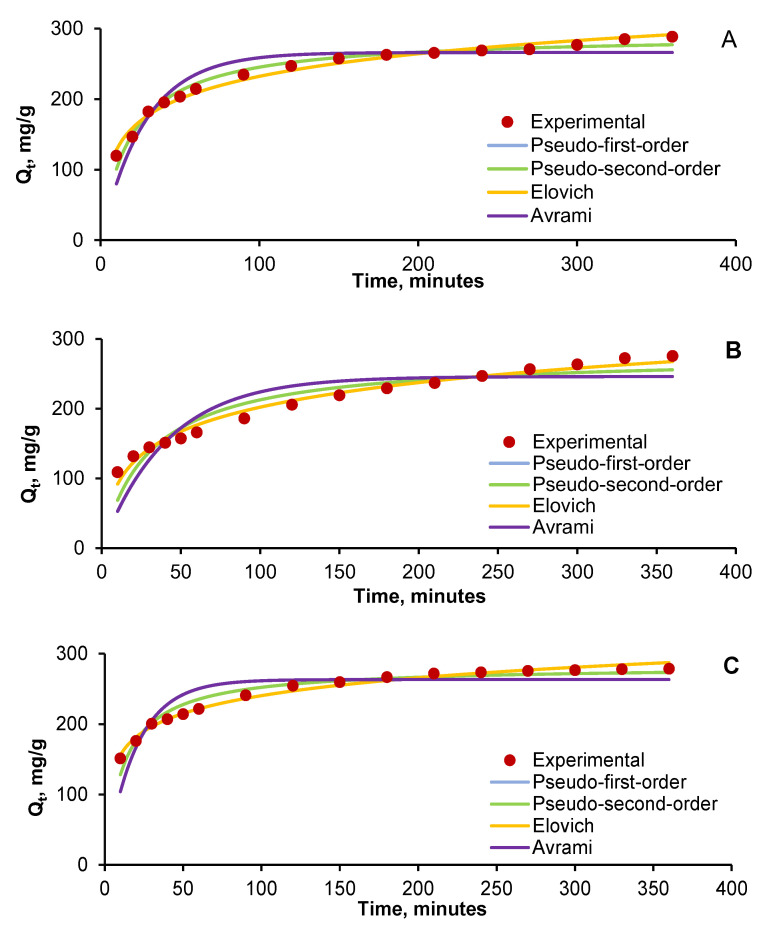
Kinetics of AR (**A**), RB (**B**), and 50% AR + 50% RB (**C**) adsorption on CSCH adsorbent.

Very similar allures were remarked for all five tested pollutant concentrations (10 mg/L, 20 mg/L, 30 mg/L, 40 mg/L, and 50 mg/L) for the two single systems (AR and RB) and the three binary systems (25% AR + 75% RB, 50% AR + 50% RB, and 75% AR + 25% RB). Therefore, we have illustrated, in Figure 7, as an example, only the kinetic behavior of the results obtained for a total initial pollutant concentration of 30 mg/L in the case of AR (Figure 7A), RB (Figure 7B), and their combination in equal proportions (Figure 7C).

From the graphical representations, it seems that the sequence Elovich > pseudo-second-order > pseudo-first-order > Avrami can be established for all cases. However, after studying the kinetic parameters values (Table 3), it can be seen that the adsorption capacities predicted by the pseudo-second-order model were closer to those of the conducted experiments.

Comparable conclusions were made by various other investigations conducted for the elimination of different combinations of dyes by adsorption on different composite materials. Rong et al. [78] analyzed the ability of an adsorbent of nickel oxide and graphene nanosheets to retain Congo Red from wastewater and indicated that the pseudo-second-order kinetic model closely fit the experimental results. Abramian and El-Rassy [79] carried out the adsorption of Orange II on titania aerogel and showed that the process follows the pseudo-second-order kinetic model. The adsorption of Reactive Black 5 dye on a low-cost adsorbent of macadamia seed husks was the subject of interest of Felista et al. [80], and their findings also designated the pseudo-second-order kinetic model as appropriate. Likewise, the adsorption of dyes existing in binary systems is well described by this type of kinetic model [81,82].

The evaluation of the statistical data (Table 4) equally endorses the indication that the Elovich and pseudo-second-order models are able to describe the contaminants adsorption on the hydrogel beads prepared by entrapping the cherry stones powder on the natural polymeric matrix of chitosan. The Elovich model fitting the experimental results implies that contaminants adsorption takes place by mass transfer with different activation energies for chemisorption. The adequacy of the pseudo-second-order equation emphasizes that chemical adsorption is the controlling mechanism of the target compounds retention, while the smaller correlation coefficients recorded for the pseudo-first-order model reveal that, even though physisorption can occur, it is not the dominant process.

### 2.4. Equilibrium Isotherms in Single and Binary Component Dye Systems

Adsorption isotherms are frequently employed for establishing the relation which occurs between the adsorption capacity reached by an adsorbent in the equilibrium state and the concentration existing initially in the pollutants aqueous solutions with the objective of determining the process mechanism.

In the present work, three isotherm models with two parameters (Langmuir, Freundlich, and Temkin) and two isotherm models with three parameters (Jovanovic-multilayer and Toth) were compared to the registered results. Langmuir isotherm characterizes the adsorption happening on adsorbent homogeneous sites. It suggests that: (i) a material possessing adsorption properties has a limited adsorption capacity; (ii) the active sites of the adsorbent are identical and can accommodate only one adsorbate molecule; (iii) there are no interactions between the retained molecules [83]. Freundlich isotherm is useful in the situation of adsorbents with heterogeneous surfaces, which retain high amounts of contaminants [84]. The Temkin equation analyses the interactions between the adsorbent and the molecule to be adsorbed. It is based on the supposition that the molecules adsorption heat diminishes in a linear mode and that the binding energies are uniformly distributed [85]. The Jovanovic-multilayer isotherm model refers to physical adsorption and implies that the adsorbent surface which is not occupied by the target compound will reduce proportionally with the decrease in the power of the compound partial pressure [86]. The Toth isotherm provides information for submonolayer coverage, its theory relying on the fact that the materials with heterogeneous surfaces are able to adsorb low-to-high concentrations of pollutants from aqueous media [87].

**Figure 8:**
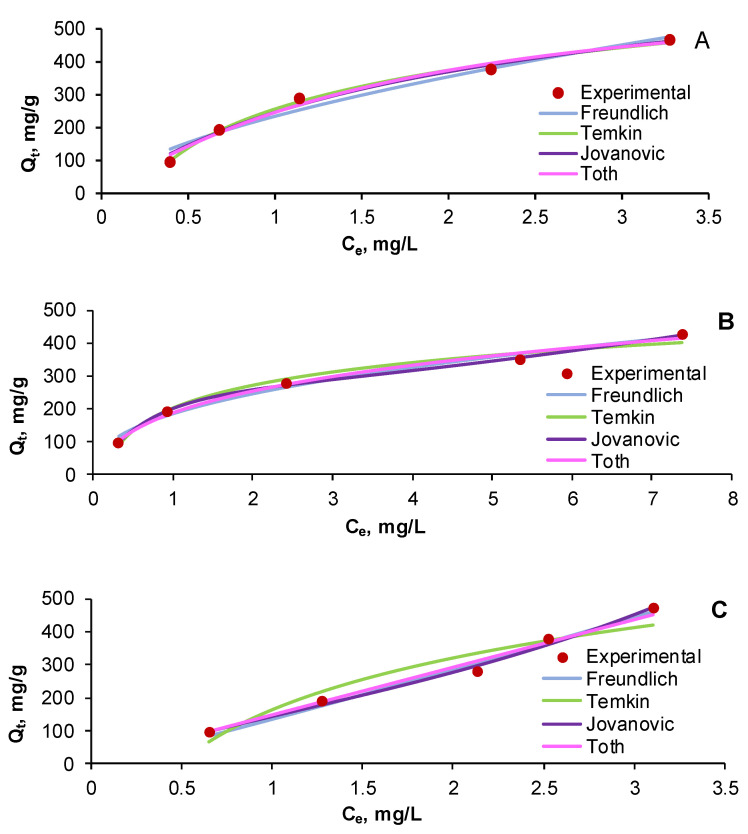
Equilibrium isotherms of AR (**A**), RB (**B**), and 50% AR + 50% RB (**C**) adsorption on CSCH adsorbent.

As in the case of the kinetic study, very similar allures were remarked for all five tested pollutant concentrations (10 mg/L, 20 mg/L, 30 mg/L, 40 mg/L, and 50 mg/L) for the two monocomponent systems (AR and RB) and for the three binary component systems (25% AR + 75% RB, 50% AR + 50% RB, and 75% AR + 25% RB). Therefore, we have pictured, in Figure 8, as an example, only the equilibrium isotherms of the results obtained for a total initial pollutant concentration of 30 mg/L in the case of AR (Figure 8A), RB (Figure 8B), and their combination in equal proportions (Figure 8C).

Regardless of the fact that Langmuir isotherm is considered one of the most frequently used equations describing the adsorption process of various types of compounds, our investigation showed that this model is not suitable for portraying the adsorption (data not shown) of chosen dyes existing in single or in binary systems.

The analysis of the equilibrium isotherms parameters, reported in Table 5, reveals that, for the monocomponent dye system RB, the adsorption is favored by temperature, as the n_F_ constant of the Freundlich isotherm is superior to 2. For both monocomponent dye systems, AR and RB, the ratio 1/n_F_ Freundlich isotherm is subunitary, suggesting a chemisorption process. For the binary component system, it is superior to 1, indicating a cooperation adsorption and, thus, a high importance of the lateral interaction among compounds on the adsorbent surface. This can be explained by the fact that the functional groups, such as hydroxyl of the prepared material, are able to form coordinative bonds. In consequence, it can be noted that the adsorbent material prepared from cherry stones powder and chitosan has characteristics that are specific to a heterogeneous surface and that the adsorption process is conducted by chemisorption and cooperative adsorption.

A good fit with experimental data can be observed for the Temkin model, its n_T_ constant suggesting a lack of adsorbent surface homogeneity.

The Jovanovic and Toth equations estimated the adsorption capacity; however, the values are rather different from those calculated experimentally, being higher in the case of the AR system and inferior for the RB system and for dye combinations.

Our conclusions are comparable with those of other studies conducted for the removal of dyes by adsorption on different materials. Similar findings were published by Ali et al. [88], who explored the adsorption of indigo carmin and Congo Red dyes on calcinated layered double hydroxides, and by Ojediran et al. [89], who inspected the retention of malachite green on acid functionalized maize cob and pointed out that the adsorption does not obey the Langmuir model. Abramian and El-Rassy focused on the adsorption of Orange II dye on a porous titania aerogel [79] and revealed that the Freundlich isotherm is suitable for describing the process. Archin et al. [90] showed that the Temkin model is applicable for the adsorption of Methylene Blue cationic dye and of Acid Blue 25 anionic dye in single form or in binary mixture on activated carbon obtained from tobacco residues. Diaz de Tuesta et al. [86] tested the removal of Sudan-IV from oily wastewater by retention on activated carbons and presented the Jovanovic and Toth expressions as appropriate models for their adsorption experiments.

The investigation of the statistical errors given in Table 6, along with the considerations discussed earlier, permit sustaining that the tested isotherm equations fit the experimental data in a satisfactory way. As a function of their adequacy and accuracy, the tried models can be ordered as follows: Freundlich > Temkin > Jovanovic > Toth.

### 2.5. Adsorption Mechanism Exploration

Depending on how the solid surface of the adsorbent interacts with the refractory compounds, the adsorption can occur by physical and/or by chemical sorption.

As explained earlier, the adsorption performance was affected by the solution pH, temperature, and adsorbent dose. The best results were obtained at pH 2, at a temperature of 30 °C, with a concentration of adsorbent of 100 g/L. In these conditions, the adsorbent is positively charged while both dyes encompass negative charges due to the existence of nitrogen–nitrogen bonds with aromatic rings, generally replaced by sulfonate groups in the case of AR, and with the sulfonate group present on the surface in the case of RB. The prepared material was able to retain the AR and RB from monocomponent systems or from binary systems based on electrostatic interactions, van der Waals forces, hydrogen, and π-π bonds. The fact that the Elovich and pseudo-second-order kinetic models were found to accurately describe the adsorption implies that chemical sorption is controlling the process. The adsorbent and the adsorbate interact mainly by strong covalent bonds. The migration of dyes from the surface to the interior of the retaining material was observable. The relation between the adsorbate and the adsorbent at equilibrium was described closely by the Freundlich and Temkin models. This information shows, also, that chemisorption and cooperative adsorption conduct the adsorption process.

## 3. Conclusions

This work focused on the synthesis of a low-cost adsorbent material based on entrapping an industrial waste represented by the cherry stones in a natural polymeric matrix such as chitosan. The resultant composite was characterized by SEM and FTIR analysis and by establishing its point of zero charge. It was then tested for its ability to adsorb emerging contaminants from aqueous solutions. Two azo dyes (Acid Red 66 and Reactive Black 5) were chosen as model molecules. Response Surface Methodology–Central Composite Design served to establish that pH 2, an adsorbent dose of 100 g/L, and a temperature of 30 °C are optimal for attaining the lowest final concentration of the mentioned dyes existing in single form or in different binary mixtures. The kinetic study carried out for various initial pollutant concentrations revealed that the adsorption mechanism is well described by the Elovich and pseudo-second-order models. In terms of equilibrium isotherms, the Freundlich and Temkin equations were found to reasonably characterize the process behavior.

The outcomes of this research allowed for confidence in the fact that the obtained hydrogel composite is a promising one and that it can be successfully utilized as an alternative to more expensive materials currently employed for removing pollutants from aqueous solutions.

## 4. Materials and Methods

### 4.1. Chemical Reagents

Analytical purity reagents were used for all the experiments without any prior treatment. All stock and diluted solutions were prepared with distilled water.

Sodium hydroxide, hydrochloric acid, acetic acid, and methanol were supplied by Chemical Company, Iasi, Romania. Glutaraldehyde was delivered by Across Organics (Vienna, Austria). Potassium nitrate, chitosan, Acid Red 66, and Reactive Black 5 dyes (Table 7) were provided by Merck, Romania.

### 4.2. Analytical Procedure

Stock solutions of Acid Red 66 and Reactive Black 5 dyes with concentrations of 100 mg/L were prepared.

When necessary, the pH was adjusted with small volumes of NaOH 0.1 M or of HCl 0.1 M. All pH measurements were made with a pH tester HI 98103 (Hanna Instruments, Bucharest, Romania).

Calibration curves in concentration range of 1 mg/L–15 mg/L for Acid Red 66 (AR) and of 1 mg/L–40 mg/L for Reactive Black 5 (RB) were used for samples evaluation. The absorbance was recorded at a wavelength of 500 nm and 600 nm for both dyes with a UV1280 Spectrophotometer (Shimadzu, Kyoto, Japan).

### 4.3. Adsorbent Synthesis

Cherry stones were recovered from fruits harvested from the Eastern Romanian region. They were washed with tap water and dried at room temperature for 24 h and in a laboratory oven (AirPerformance AP60, Froilabo, Paris, France) at 60 °C for 6 h. Then, they were passed through a grinding mill (PerkinElmer, Hägersten, Sweden) and sieved on a 125-µm sieve existing on an electromagnetic Filtra Vibracion IRIS FTS-0200 sieve shaker (Filtra Vibracion, Badalona, Spain).

The adsorbent was prepared based on the method proposed by Altun et al., 2019 [58] with slight modifications. Briefly, 4 g of chitosan were put in contact with 200 mL of a 2% acetic acid solution on a Nahita magnetic plate (Auxilab, Beriáin, Spain). After dissolution, 2 g of cherry stone powder were added and continuously stirred for 24 h. The resultant mixture was dropped into a coagulation solution composed of 120 g of NaOH, 400 mL of distilled water, and 600 mL of methanol. The obtained hydrogel beads were let in this solution for 24 h in order to insure their complete coagulation. After that, they were washed with distilled water until they had a neutral pH and then placed in a laboratory beaker along with a solution containing 1.2 mL glutaraldehyde and 120 mL methanol, and were heated at reflux at 70 °C for 6 h. The prepared cherry stones-chitosan hydrogel beads (abbreviated CSCH) were stored in glutaraldehyde-methanol solution in a closed vessel at 4 °C. Prior to use, they were washed with ethanol and distilled water.

### 4.4. Adsorbent Characterization

Before SEM analysis, the adsorbent was dried overnight at room temperature. Then, it was positioned on double-adhesive carbon discs fixed on specific stubs of a TESCAN MIRA device (TESCAN Orsay Holding, Brno, Check Republic) equipped with TESCAN Essence software version 1.0.8.0. Normal secondary electron mode (SE) in high vacuum was considered for the investigation. Detection was ensured by a large field detector (LFD) at an accelerating voltage of 20 kV, a working distance of 14–40 mm, and a spot size of 6. The magnification range was between 500 μm and 50 µm.

FTIR spectra were registered from 4000 cm^−1^ to 400 cm^−1^ (45 scans/min.) with a resolution of 4 cm^−1^ on an IRSpirit FT-IR spectrometer (Shimadzu, Bucharest, Romania) including a QATR accessory and a KBr beam splitter. The QATR plate was cleaned with ethanol between acquisitions. Air was used for background spectrum reference, which was recorded and compared with the anterior one.

Determination of pH_PZC_ was realized by solid addition method with potassium nitrate as background electrolyte following the procedure described by Balistrieri and Murray [91]. Summarily, volumes of 25 mL of KNO_3_ 0.1 M were added in a series of Erlenmeyer flasks. An adjustment between 2 and 12 of initial pH values (pH_i_) was ensured. In each initial solution, 0.5 g of CSCH beads were added. After 24 h at room temperature, the solutions pH was measured again (pH_f_). A graphical representation of the evolution of differences between the initial and final pH values (Δ pH) against pH_i_ served to establish the pH_PZC_ of the samples.

### 4.5. Optimization of Adsorption Process Main Parameters

Based on preliminary tests, three independent variables and one response function were chosen for the study. The coded and actual values of the considered factors included in the experimental matrix design are presented in Table 8.

10 mL of AR solution, 10 mL of RB solution, or 10 mL of a mixture containing 50% AR and 50% RB with a concentration of 30 mg/L with variable pH were put in contact with different amounts of cherry stones-chitosan hydrogel beads at various temperatures for 6 h.

The final dye concentrations were determined by reading the absorbance at 500 nm for AR and at 600 nm for RB. For the binary mixture, the absorbance was recorded both at 500 nm and at 600 nm. To establish the concentration in this case, Vierordt’s method (or the simultaneous equation method) was applied using Equations (4) and (5).

(4) $$C_{\mathrm{AR}}=\frac{A_{\lambda 1}\cdot \varepsilon_{\mathrm{RB}}^{\lambda 1}-A_{\lambda 2}\cdot \varepsilon_{\mathrm{RB}}^{\lambda 2}}{\varepsilon_{\mathrm{AR}}^{\lambda 1}\cdot \varepsilon_{\mathrm{RB}}^{\lambda 2}-\varepsilon_{\mathrm{RB}}^{\lambda 1}\cdot \varepsilon_{\mathrm{AR}}^{\lambda 2}}$$

(5) $$C_{\mathrm{RB}}=\frac{A_{\lambda 1}\cdot \varepsilon_{\mathrm{RB}}^{\lambda 1}-A_{\lambda 2}\cdot \varepsilon_{\mathrm{RB}}^{\lambda 2}}{\varepsilon_{\mathrm{AR}}^{\lambda 1}\cdot \varepsilon_{\mathrm{RB}}^{\lambda 2}-\varepsilon_{\mathrm{RB}}^{\lambda 1}\cdot \varepsilon_{\mathrm{AR}}^{\lambda 2}}$$

where C_AR_ and C_RB_ are the concentrations (mg/L) of AR and RB; A_λ1_ is the absorbance of the mixture at 500 nm; A_λ2_ is the absorbance of the mixture at 600 nm; ε_RB_^λ1^ and ε_RB_^λ2^ are the molar absorptivities of RB at 500 nm and 600 nm, respectively; ε_AR_^λ1^ and ε_AR_^λ2^ are the molar absorptivities of AR at 500 nm and 600 nm, respectively. Molar absorptivities were represented by the slopes of the calibration curves.

All experiments were carried out in triplicate and reported as mean values.

The obtained data were introduced in Design Expert 13 software (Stat-Ease, Minneapolis, MN, USA) and Response Surface Methodology–Central Composite Design (RSM-CCD) was used to find the optimum values of the working parameters and to generate quadratic polynomial models expressed by Equation (6):

(6) $$Y_{K}=\beta_{0}+\overset{n}{\underset{i=1}{\sum }}\beta_{i}\cdot X_{i}+\overset{n}{\underset{i=1}{\sum }}\overset{n}{\underset{j=1}{\sum }}\beta_{\mathrm{ij}}\cdot X_{i}\cdot X_{j}+\overset{n}{\underset{i=1}{\sum }}\beta_{\mathrm{ii}}\cdot X_{i}^{2}$$

where Y_K_ is the response function (final dye concentration, mg/L), X_i_ stands for independent variables, β_0_, β_i_, β_j_, and β_ij_ are the intercept, linear, quadratic, and interaction coefficients.

Removal efficiency (R, %) and equilibrium sorption capacity were calculated with Equations (7) and (8):

(7) $$R=\frac{C_{i}-C_{e}}{C_{i}}\cdot 100$$

(8) $$q_{e}=\frac{C_{i}-C_{e}}{M}\cdot V$$

where C_i_ and C_e_ are the initial and equilibrium concentrations (mg/L); V is the dye volume (mL); M is the adsorbent amount (g).

### 4.6. Kinetic Study and Equilibrium Isotherms

In the established optimal conditions, 10 mL of AR, of RB, or of AR + RB combinations with concentrations between 10 mg/L and 50 mg/L were mixed with the adsorbent. The pollutant retention was followed between 10 min and 240 min.

Each of the kinetic and equilibrium isotherm models has its own nonlinear equation. Their forms and related explanations are shown in Table 9 and Table 10. Experimental data were explored with the help of CAVS software, version 2.0 (Federal University of Paraná, Curitiba, Paraná, Brazil).

### 4.7. Statiscal Analysis

The results obtained by carrying out the experimental program concentrated on the elimination of dyes from aqueous solutions by adsorption on the prepared adsorbent were statistically evaluated by Analysis of Variance (ANOVA) (in the case of RSM), Root mean square error (RMSE), Marquardt’s percent standard deviation (MPSD), hybrid fractional error function (HYBRID), chi-square (χ^2^), and coefficient of determination (R^2^) (in the case of the kinetic and equilibrium isotherms study). All the values were generated by the software specified in Section 4.5 and Section 4.6.

## Figures and Tables

**Table 1:** RSM-CCD matrix including experimentally obtained and model-predicted data.

Run	Variables			Final Contaminant Concentration, mg/L					
	X_1_	X_2_	X_3_	AR		RB		50% AR + 50% RB	
				Observed	Predicted	Observed	Predicted	Observed	Predicted
1	2	50	20	8.07	7.48	9.79	9.84	18.12	16.37
2	2	50	30	7.16	6.54	7.71	7.69	17.29	16.25
3	2	50	40	8.07	7.53	9.67	9.57	18.81	17.48
4	2	100	20	2.08	2.19	4.63	4.45	9.65	11.06
5	2	100	30	1.14	1.58	2.40	2.39	7.07	10.98
6	2	100	40	1.84	2.90	4.55	4.37	11.23	12.26
7	2	150	20	3.75	3.97	6.73	6.94	14.22	14.31
8	2	150	30	3.47	3.68	4.92	4.98	16.65	14.28
9	2	150	40	5.60	5.32	6.87	7.06	15.56	15.60
10	7	50	20	14.37	15.39	18.20	18.23	22.52	23.76
11	7	50	30	13.65	14.15	15.92	15.89	22.12	23.39
12	7	50	40	14.19	14.85	17.26	17.58	22.78	24.37
13	7	100	20	9.08	9.45	12.82	12.94	20.54	19.22
14	7	100	30	8.78	8.54	10.24	10.69	19.80	18.89
15	7	100	40	10.32	9.57	12.62	12.48	22.12	19.91
16	7	150	20	11.49	10.57	15.88	15.53	23.34	23.23
17	7	150	30	10.22	9.99	13.73	13.38	22.97	22.95
18	7	150	40	11.77	11.34	15.32	15.27	23.57	24.02
19	12	50	20	22.43	21.90	26.59	26.33	26.97	27.09
20	12	50	30	20.36	20.38	23.75	23.80	26.59	26.47
21	12	50	40	20.73	20.79	25.32	25.30	27.18	27.20
22	12	100	20	15.40	15.32	21.00	21.14	23.73	23.31
23	12	100	30	14.57	14.12	18.57	18.70	23.70	22.73
24	12	100	40	15.31	14.86	20.64	20.30	24.03	23.50
25	12	150	20	15.40	15.79	23.60	23.84	27.35	28.09
26	12	150	30	14.56	14.92	21.77	21.50	27.33	27.56
27	12	150	40	15.31	15.98	22.86	23.19	27.44	28.37

**Table 2:** Analysis of variance for adsorption of dyes in single or binary system on the synthesized adsorbent material.

Source	Sum of Squares	df	Mean Square	*F*-Value	*p*-Value
**AR**					
Model	890.28	9	98.92	260.08	<0.0001
X_1_—pH	707.86	1	707.86	1861.08	<0.0001
X_2_—Adsorbent dose	77.92	1	77.92	204.86	<0.0001
X_3_—Temperature	0.0642	1	0.0642	0.1687	0.6857
X_1_X_2_	5.06	1	5.06	13.31	0.0016
X_1_X_3_	1.02	1	1.02	2.67	0.1177
X_2_X_3_	1.28	1	1.28	3.38	0.0811
X_1_^2^	3.76	1	3.76	9.89	0.0051
X_2_^2^	83.08	1	83.08	218.44	<0.0001
X_3_^2^	5.76	1	5.76	15.15	0.0009
Residual	7.61	20	0.3803		
Lack of Fit	7.60	17	0.4472	254.31	0.0004
Pure Error	0.0053	3	0.0018		
Cor Total	897.89	29			
**RB**					
Model	1383.54	9	153.73	1877.61	<0.0001
X_1_—pH	1197.72	1	1197.72	14,628.93	<0.0001
X_2_—Adsorbent dose	28.20	1	28.20	344.43	<0.0001
X_3_—Temperature	0.9476	1	0.9476	11.57	0.0028
X_1_X_2_	0.1240	1	0.1240	1.51	0.2327
X_1_X_3_	0.4447	1	0.4447	5.43	0.0304
X_2_X_3_	0.1141	1	0.1141	1.39	0.2517
X_1_^2^	0.1900	1	0.1900	2.32	0.1433
X_2_^2^	105.38	1	105.38	1287.11	<0.0001
X_3_^2^	27.34	1	27.34	333.96	<0.0001
Residual	1.64	20	0.0819		
Lack of Fit	0.9900	17	0.0582	0.2698	0.9678
Pure Error	0.6475	3	0.2158		
Cor Total	1385.18	29			
**50% AR + 50% RB**					
Model	767.58	9	85.29	34.43	<0.0001
X_1_—pH	620.92	1	620.92	250.64	<0.0001
X_2_—Adsorbent dose	0.8736	1	0.8736	0.3526	0.5593
X_3_—Temperature	2.19	1	2.19	0.8849	0.3581
X_1_X_2_	7.01	1	7.01	2.83	0.1080
X_1_X_3_	0.7594	1	0.7594	0.3065	0.5859
X_2_X_3_	0.0215	1	0.0215	0.0087	0.9266
X_1_^2^	35.51	1	35.51	14.34	0.0012
X_2_^2^	111.81	1	111.81	45.13	<0.0001
X_3_^2^	1.28	1	1.28	0.5163	0.4807
Residual	49.55	20	2.48		
Lack of Fit	48.87	17	2.87	12.65	0.0295
Pure Error	0.6819	3	0.2273		
Cor Total	817.13	29			

**Table 3:** Kinetic parameters of adsorption of AR, RB, and 50% AR + 50% RB.

Kinetic Model	Parameters	Dye System		
		AR	RB	50% AR + 50% RB
Pseudo-first-order	Q_e_	266.0630	246.2734	263.1561
	k_1_	0.0357	0.0241	0.0502
Pseudo-second-order	Q_e_	291.7014	277.7586	282.5912
	k_2_	0.0001	0.0001	0.0002
Elovich	α	68.9711	25.4416	46.8997
	β	0.0215	0.0193	0.0008
Avrami	Q_e_	266.0640	246.2756	263.1573
	k_Av_	0.0048	0.0037	0.0097
	n_Av_	7.3976	6.5119	5.1388

**Table 4:** Statistical error function values of kinetic nonlinear models.

Kinetic Model	Statistical Error	Dye System		
		AR	RB	50% AR + 50% RB
Pseudo-1st-order	RMSE	16.3835	24.9997	19.0718
	MPSD	10.7211	18.4442	10.0718
	HYBRID	176.581	468.3276	216.2806
	Χ^2^	31.1769	99.9418	36.2142
	R^2^	0.8889	0.7755	0.7631
Pseudo-2nd-order	RMSE	7.1010	16.9111	8.8147
	MPSD	4.8352	12.6683	5.1598
	HYBRID	34.4764	217.7377	47.5154
	Χ^2^	5.3919	39.4300	7.2024
	R^2^	0.9791	0.8972	0.9494
Elovich	RMSE	6.0496	7.8381	4.6171
	MPSD	3.6209	5.7136	2.1060
	HYBRID	22.3955	45.6905	10.1622
	Χ^2^	3.0401	6.8212	1.4145
	R^2^	0.9848	0.9779	0.9861
Avrami	RMSE	16.3834	24.9997	19.0718
	MPSD	11.1263	19.1411	11.2905
	HYBRID	190.1722	504.3717	232.9243
	Χ^2^	31.1797	99.9527	36.2170
	R^2^	0.8888	0.7755	0.7631

**Table 5:** Equilibrium isotherm parameters of adsorption of AR, RB, and 50% AR + 50% RB.

Equilibrium Isotherm	Parameters	Dye System		
		AR	RB	50% AR + 50% RB
Freundlich	K_F_	234.4442	183.2031	134.9317
	n_F_	1.6768	2.4246	0.9236
Temkin	K_T_	4.5322	25.5301	11.1164
	b_T_	14.8290	7.6888	2.0320
Jovanovic-multilayer	Q_J_	356.0906	222.6863	120.8938
	K_J_	1.0000	1.7227	1.4182
	K’_J_	0.0917	0.0867	0.4418
Toth	Q_To_	1157.282	226.8143	145.5048
	K_To_	3.0521	0.2571	3.8275
	n_To_	1.1041	0.8255	0.00006

**Table 6:** Statistical error function values of equilibrium isotherm nonlinear models.

Equilibrium Isotherm	Statistical Error	Dye System		
		AR	RB	50% AR + 50% RB
Freundlich	RMSE	23.8625	14.8366	14.9614
	MPSD	24.4571	12.0034	8.6964
	HYBRID	676.8724	198.3764	153.0725
	Χ^2^	16.3826	5.5098	4.4373
	R^2^	0.9668	0.9834	0.9872
Temkin	RMSE	9.5809	16.8941	38.2854
	MPSD	3.8981	7.6475	24.0721
	HYBRID	44.5042	145.4721	1004.89
	Χ^2^	1.3125	4.4617	32.6841
	R^2^	0.9946	0.9785	0.9167
Jovanovic-multilayer	RMSE	16.6873	4.7557	11.2649
	MPSD	19.0802	2.3253	6.9566
	HYBRID	418.4485	17.2520	115.684
	Χ^2^	7.1594	0.3431	2.2864
	R^2^	0.9856	0.9983	0.9927
Toth	RMSE	16.0543	12.7540	16.6615
	MPSD	17.4819	7.9484	8.8027
	HYBRID	379.7791	140.7021	227.6069
	Χ^2^	6.7054	2.7508	4.1725
	R^2^	0.9850	0.9877	0.9842

**Table 7:** Model azo dyes properties.

Properties	Acid Red 66	Reactive Black 5
Structure	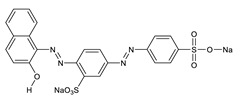	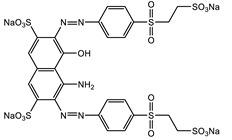
Chemical name	Sodium 6-(2-hydroxynaphthylazo)-3,4′-azodibenzenesulfonate	Tetrasodium 4-amino-5-hydroxy-3,6[[4-[[2-(sulphonatooxy)ethyl]supphonyl]pehnyl]azo]naphthalene-2,7-disulphonate
Molecular formula	C_22_H_14_N_4_Na_2_O_7_S_2_	C_26_H_21_N_5_Na_4_O_19_S_6_
Molecular weight	556.48 g/mol	991.82 g/mol
CAS Number	4196.99-0	17095-24-8
EC Number	224-084-5	241-164-5
Maximum wavelength	500 nm	600 nm

**Table 8:** Variables and levels of variation in RSM-CCD.

Variable		Levels of Variation		
Coded	Uncoded	Low (−1)	Central (0)	High (+1)
X_1_	pH	2	7	12
X_2_	Adsorbent dose, mg/L	50	100	150
X_3_	Temperature, °C	20	30	40

**Table 9:** Kinetic models nonlinear equations.

Kinetic Model	Equation	Parameters Significance *
Pseudo-1st-order	$Q_{t}=Q_{e}\cdot \left(1-e^{-k_{1}\cdot t}\right)$	k_1_—pseudo-first-order constant rate, min^−1^
Pseudo-2nd-order	$Q_{t}=\frac{k_{2}\cdot Q_{e}^{2}\cdot t}{\left(1+k_{2}\cdot Q_{e}\cdot t\right)}$	k_2_—pseudo-2nd-order constant rate, g/(mg·min)
Elovich	$Q_{t}=\frac{1}{\beta }\cdot \ln\left(\alpha \cdot \beta \cdot t+1\right)$	β—extent of surface coverage and activation energy for chemisorption, g/mg α—initial adsorption rate, mg/(g·min)
Avrami	$Q_{t}=Q_{e}\cdot \left(1-e^{\left(-k_{\mathrm{Av}}\cdot t{)}^{n_{\mathrm{Av}}}\right)}\right)$	k_Av_—global rate constant, min^−1^ n_Av_—factor related to adsorption, dimensionless

* Common symbols significance: Q_t_—concentration on the solid phase at time t, mg/g; Q_e_—adsorbent capacity at equilibrium, mg/g; t—contact time, min.

**Table 10:** Equilibrium isotherms nonlinear equations.

Equilibrium Isotherm	Equation	Parameters Significance *
Langmuir	$Q_{e}=\frac{Q_{L}\cdot K_{L}\cdot C_{e}}{1+K_{L}\cdot C_{e}}$	Q_L_—maximum Langmuir uptake, mg/g K_L_—Langmuir constant, L/mg
Freundlich	$Q_{e}=K_{F}\cdot C_{e}^{1/n_{F}}$	K_F_—Freundlich constant, (mg/g)(L/mg)^1/n^ n_F_—Freundlich constant, dimensionless
Temkin	$Q_{e}=\frac{R\cdot T}{b_{T}}\cdot \ln\left(K_{T}\cdot C_{e}\right)$	R—gas constant, R = 8.314 J/(mol K) T—temperature, K K_T_—Temkin constant, L/mg b_T_—Temkin constant, J/mg
Jovanovic-multilayer	$Q_{e}=Q_{J}\cdot (1-e^{-K_{J}\cdot C_{e}}\cdot e^{-K_{J}^{\prime }\cdot C_{e}})$	Q_J_—Jovanovic maximum uptake, mg/g K_J_—Jovanovic constant, L/mg K’_J_—Jovanovic constant, L/mg
Toth	$Q_{e}=\frac{Q_{\mathrm{To}}\cdot C_{e}}{{\left(\frac{1}{K_{\mathrm{To}}}+C_{e}{}^{n_{\mathrm{To}}}\right)}^{1/n_{\mathrm{To}}}}$	Q_To_—Toth maximum uptake, mg/g K_To_—Toth constant, L/mg n_To_—Toth constant, dimensionless

* Common symbols significance: Q_e_—adsorbate concentration on solid phase at equilibrium, mg/g; C_e_—adsorbate concentration on fluid phase at equilibrium, mg/L.

## Data Availability

Not applicable.
